# Flavonoid Contents and Free Radical Scavenging Activity of Extracts from Leaves, Stems, Rachis and Roots of *Dryopteris erythrosora*

**Published:** 2012

**Authors:** Min Zhang, Jianguo Cao, Xiling Dai, Xuefei Chen, Quanxi Wang

**Affiliations:** Department of Biology, College of Life and Environment Sciences, Shanghai Normal University, Guilin Rd, Shanghai, China.

**Keywords:** Flavonoids, Free radical scavenging, Extracts, *Dryopteris erythrosora*

## Abstract

The present study was designed to explore the flavonoid contents and the radical scavenging activities of 50% ethanol extracts of leaves, stems, rachis and roots of Dryopteris erythrosora. The total flavonoid contents in various parts were determined as: stems > roots > rachis > leaves. All extracts from different parts of D. erythrosora showed strong bioactivities. The DPPH free radical scavenging abilities of extracts were determined as: stems > root > rachis >> leaves. This trend is reciprocal proportion to the total flavonoid contents in leaves, stems, rachis and roots of D. erythrosora extracts. The superoxide anion scavenging abilities of ethanol extracts were determined as: stems > leaves > rachis > root. It illustrated that there are strong superoxide anion scavengers in D. erythrosora. It is worth to separate and identify these components in future.

## Introduction

Fern plants are well-known traditional Chinese medicinal herbs and extensively used to treat the skin tumefaction, to protect the liver and to treat the hepatitis and also being used as antipyretics (1). Large numbers of ferns such as *Dryopteris erythrosora*, *Cyrtomium fortunei*, and *Coniogramme japonica *occur in Jiangsu and Zhejiang provinces (2).

The Autumn Fern (*D. erythrosora*) is native to China and Japan, but its common name is “Japanese Red Shield Fern” because of its youngest leaves appearing to coppery red to bronze or pink throughout the spring. The most often-used common name “Autumn Fern” is for the very reason of its colorfulness, though it has these colors more in spring rather than in autumn.

The major identified constituents in *Dryopteris *plants are flavonoids, polyphenols and terpenoids (3). Ten flavonol *O*-glycosides (based on kaempferol and quercetin), two flavanone *O*-glycosides (based on naringenin and eriodictyol) and three *C*-glycosylflavones (vitexin, vitexin 7-*O*-glucoside and orientin) in eighteen *Dryopteris *species have previously been identified by Hiraoka (4). The 3-Desoxyanthocyanins have been found in *D. erythrosora *by Harborne (5). In addition, kaempferol 7-*O*-(6″-succinyl-glucoside) was found in four *Dryopteris *species and an unusual flavan was isolated from *D. filix-mas *(L.) Schott (6). Three new kaempferol glycosides, namely kaempferol 3-*α*-L-(2,4-di-*O*-acetyl) rhamnopyranoside-7-*α*-Lrhamnopyranoside, kaempferol 3-*α*-L-(3,4-di-*O*-acetyl) rhamnopyranoside-7-*α*- L-rhamnopyranoside and kaempferol 3-*α*-L-(2,3-di-*O*-acetyl) rhamnopyranosside-7-*α*-L-rhamnopyranoside were isolated from the rhizome of *D. crassirhizoma *(Aspidiaceae) (7). Ten flavonoids (seven flavonol glycosides based on kaempferol and quercetin including a new compound identified as kaempferol 3-O-(acetylrutinoside) and three flavonoid aglycones (apigenin, kaempferol and quercetin)) were found in *D. villarii *(Bell.) Woynar by Imperato *et al. *(8). Recently, Imperato have found five new flavonoids, quercetin 3-O-(X»-acetyl-X″-cinnamoyl-glucoside), quercetin 3-O- (glucosyl-rhamnoside), Kaempferol 3-O-(caffeoylrhamnoside), apigenin 4›-O- (caffeoylglucoside) and 4›-O-(feruloylglucoside) from *D. villarii *(9-10).

However, there is little information about the flavonoids of *D. erythrosora*. Chang *et al. *reported that the total flavonoids content in *D. erythrosora *were about 0.89% (2). Flavonoids are widely existed in the plant kingdom, with a listing of 6467 known structures (11-15). Flavonoids are of great interest for their bioactivities, which are basically related to their antioxidant properties (16-21). Herein, the flavonoid contents and free radical scavenging activities of extracts from leaves, stems, rachis and roots of *D. erythrosora *were investigated.

## Experimental

*Chemicals and materials*

Rutin (> 98%) and DPPH were purchased from Sigma Co. (MO, USA). Biochanin A, apigenin, catechin and luteolin (99.0%) were purchased from Aladdin Co. Ltd. (Shanghai, China). The 7-Hydroxyflavanone was purchased from TCI Chemical Industries (Tokyo, Japan). Other flavonoid standards were obtained commercially from Shanghai Tauto Biotech CO., Ltd (Shanghai, China). Nitrotetrazolium blue chloride (NBT), phenazine methosulfate (PMS) and 2,2-Diphenyl-1-picrylhydrazyl (DPPH) were purchased from Aladdin Reagent Int. (Shanghai, China). Nicotinamide adenine dinucleotide (NADH) was obtained from Sangon Biotech (Shanghai) Co. Ltd. D. erythrosora was obtained from Sheshan in Shanghai, China. The identities of the plants were verified by Dr. Jianguo Cao in College of Life and Environment Sciences of Shanghai Normal University. Acetonitrile was of HPLC grade. All other reagents and solvents were of analytical grade and all aqueous solutions were prepared using newly double distilled water.

*Preparation of plant extracts*

The fresh leaves, stems, rachis, and roots of *D. erythrosora *were collected, washed, and dried in shade. The dried sample was powdered and filtered through 40-mesh screen. The dried sample (2.0000 g) was extracted with 50% ethanol (25 mL) for 5 h at room temperature. Then, the ultrasound-assisted extraction was performed on a Kunshan ultrasound generation system for 20 min. This extraction process was repeated twice for each sample. The extracts were filtered with filter paper and then collected. The mixture was allowed to cool for 20 min and concentrated through a rotary evaporator for the purpose of getting dry. The residue was suspended with 50 mL methanol and filtered through a 0.45 μm membrane (Millipore, USA) before the test.

*Determination of flavonoids*

*Total flavonoids*

The total flavonoid content was measured through a colorimetric assay. The extract (5 mL) was added to a 10 mL flask and then 5% NaNO_2_ solution (0.3 mL) was added to it. After mixing well, the solution was allowed to stand for 6 min at room temperature and 5% Al(NO_3_)_3_ solution (0.3 mL) was added to the flask, mixed well and kept for 6 min at room temperature. At last, 4% NaOH solution (4.4 mL) was added, mixed well and kept for 12 min at room temperature. The absorbance was read on a *TU-1810 UV-*spectrophotometer (Beijing, China) at 510 nm and the flavonoid percentage was estimated using the calibration curves.

*HPLC analysis*

HPLC analysis was performed on a Waters 600 apparatus. The separation was carried out on a TSKgel ODS-100Z C18 column (5 μm, 250 × 4.6 mm i.d.). The elution was carried out with a gradient solvent system with a flow rate of 1.0 mL/min at 25°C. The mobile phase consisted of acetonitrile (A) and 0.3% CH_3_COOH (B). The qualitative levels were determined using the above solvents programmed linearly from 10 to 50% A for 0-20 min. The gradient then kept 50% A for 15 min. The detection wavelength was 280 nm. The injection volume was 20 μL.

*DPPH free radical scavenging activity*

Spectrophotometric analyses were recorded on a *TU-1810 UV-*spectrophotometer made by Puxi Tongyong (Beijing, China) to determine the DPPH free radical scavenging ability. The effects of *D. erythrosora *extracts on free radical scavenging were assayed according to the references (22-23). Two milliliters of a freshly prepared DPPH solution (100 μmol/L) in ethanol was placed in a cuvette and the extracts solution was added with a different volume. After 30 min of incubation at room temperature in the dark, the absorbance of the mixture was recorded at 515 nm against a second cuvette with a blank solution of DPPH.

*Superoxide anion scavenging activity*

Superoxide anion scavenging activity was measured according to the reference (24) and slightly modified. In this experiment, the superoxide radical was generated in 3 mL of sodium phosphate buffer (100 mM, pH = 7.4) containing 1 mL of NBT solution (150 μM), 1 mL of NADH solution (468 μM) and different concentrations of *D. erythrosora *extracts (25-400 μg/mL) in methanol. The reaction started with adding 1 mL of PMS (60 μM) to the mixture. The reaction mixture was incubated at 25°C for 5 min and the absorbance was measured at 560 nm. Compared with the value with no test sample added, the reduction of the absorbance was estimated as superoxide scavenging activity. The capability of scavenging the superoxide radical was calculated using the following equation:

Superoxide anion scavenging activity (%) = (1 - A_0_ / A_1_) × 100

Here, *A*_0 _is the absorbance of the control, only without sample and *A*_1_ is the absorbance of sample.

## Results and Discussion

*Total flavonoid content in different parts of D. erythrosora*

**Figure 1:**
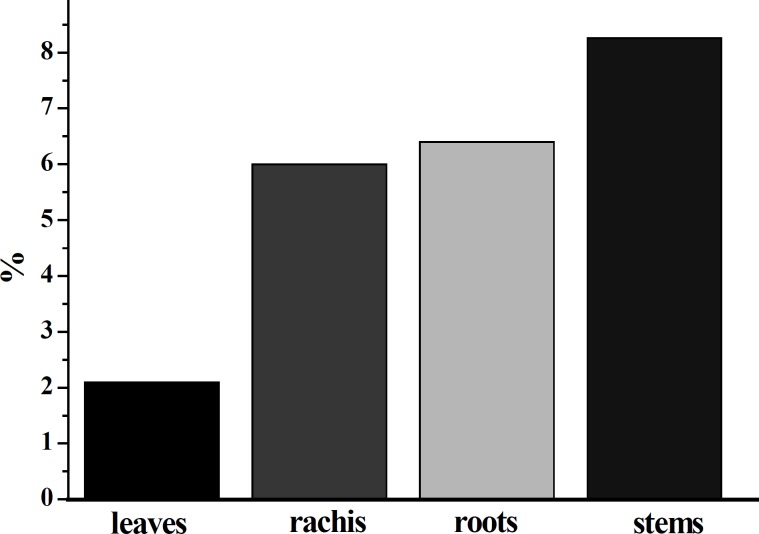
The total flavonoid contents in leaves, stems, rachis and roots of *D. erythrosora *were ranged from 2.1% to 8.26%.

The 50% ethanol was used to extract the flavonoids from fresh leaves, stems, rachis and roots of *D. erythrosora *according to the above procedure. As shown in Figure 1, the total flavonoid contents in leaves, stems, rachis and roots of *D. erythrosora *were ranged from 2.1% to 8.26%. The total flavonoid contents in various parts were determined as: stems > roots > rachis > leaves. The total flavonoid contents in stems (8.26%), roots (6.4%) and rachis (6%) were much higher than that in leaves (2.1%). *Chang et al. *found that the total flavonoid content in the leaves of *D. erythrosora *was about 0.89% (2). Here, the total flavonoid content in leaves was about 2.1%, which was similar to the above reference. Fang *et al. *determined the total flavonoids contents in 32 kinds of fern plants in Yunnan Province of China and found that the total flavonoid contents were within the range of 0.50-8.70% (25). The total flavonoid contents in *Dryopteris *plants were within the range of 3.58-5.88%.

*Flavonoids distribution in D. erythrosora*

Some representatives of flavonoids were detected in different parts of *D. erythrosora *by means of HPLC with relative retention time. The flavonoids distribution in *D. erythrosora *was shown in Table 1. As shown in Table 1, the flavonoid distribution in different parts of *D. erythrosora *was significantly different. Only dihydromyricetin was detected in the roots and found in all parts of *D. erythrosora*. However, more than ten kinds of flavonoids were found in the rachis.

**Table 1:** The flavonoids distribution in *D. erythrosora*

**Flavonoids**	**Roots**	**Stems**	**Leaves**		**Rachis**
**Dihydromyriceti** **n**	+	+	+		+
**7-hydroxyflavone**	–	–	+		–
**Apigenin**	–	–	–		+
**Luteolin**	–	+	–		+
**Kaempferide**	–	+	–		+
**Biochanin A**	–	+	–		–
**Dadzein**	–	+	–		–
**Dadzin**	–	–	+		+
**Tectorigenin**	–	–	+		+
**Baicalin**	–	–		+	
**Nobiletin**	–	–		+	
**Wogonin**	–	–	+		+
**Formononetin**	–	–	+		–
**Catechin**	–	–	–		+
**Galangin**	–	–	–		–
**Kaempferol**	–	–	–		–
**Quercetin**	–	–	–		–
**Myricetin**	–	–	–		–
**Rutin**	–	–	–		–

“+” means found; “-” means not found

In a survey of 15 representatives of the *Asplenioid *ferns for the pinnae flavonoids, the kaempferol, quercetin and proanthocyanidin were found in 80%, 53% and 13% of the studied species, respectively (26). Harborne found 3-desoxyanthocyanins in *D. erythrosora *(5). However, kaempferol and quercetin were not detected in all parts of *D. erythrosora*. The further work will focus on the new flavonoids in *D. erythrosora.*

*DPPH radical scavenging activity of different parts of D. erythrosora*

**Figure 2:**
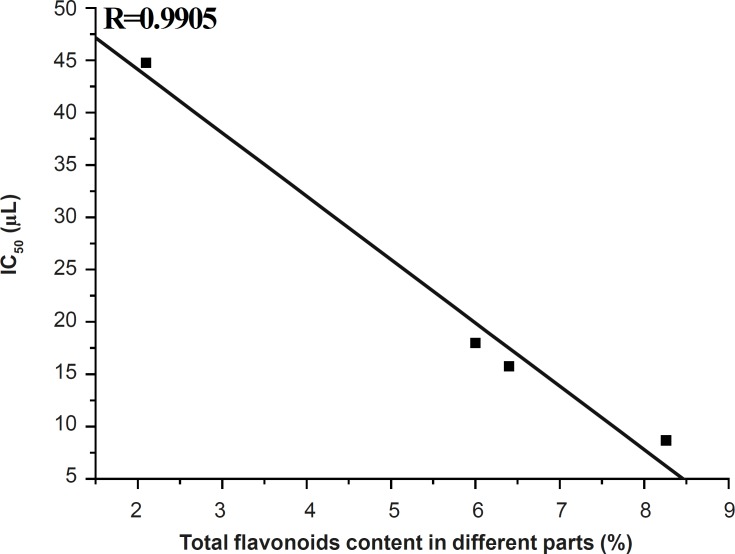
The DPPH scavenging activities of 50% ethanol extracts from different parts of *D. erythrosora*. The DPPH free radical scavenging abilities of extracts were determined as: stems > root > rachis >> leaves

DPPH assay is based on the measurement of the scavenging ability of antioxidants towards the stable radical DPPH. It is considered a valid and easy assay to evaluate the radical-scavenging activity (RSA) of antioxidants. The DPPH scavenging activities of 50 ethanol extracts from different parts of *D. erythrosora *were shown in Figure 2. 

**Figure 3:**
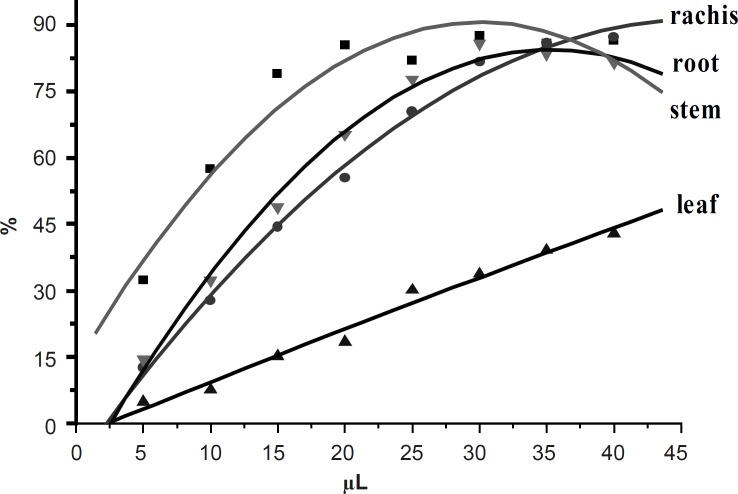
The linear relationship between DPPH free radical scavenging abilities and total flavonoids contents

The DPPH scavenging activities of stems, root and rachis extracts were increased with the increase of volumes from 5-20 μL and hardly changed after 25 μL. However, the DPPH scavenging activities of leaves extracts continually increased. The DPPH free radical scavenging abilities of extracts were determined as: stems > root > rachis >> leaves. This trend is a reciprocal proportion to the total flavonoids contents in leaves, stems, rachis and roots of *D. erythrosora*

 (Figure 3). The stems extract showed very high DPPH radical scavenging activities in the range of 79.0%-87.6% at 15-45 μL. The leaves extract showed the lowest DPPH radical scavenging activities.

*Superoxide anion scavenging activity of different parts of D. erythrosora*

**Figure 4:**
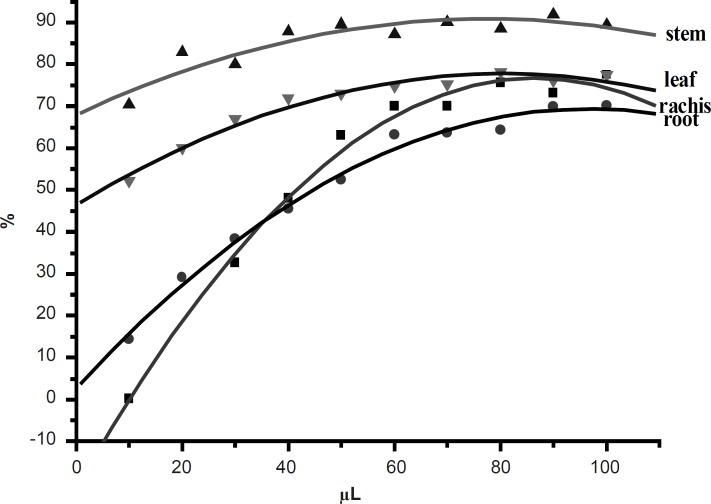
The superoxide anion scavenging activities of 50% ethanol extracts from different parts of *D. erythroso ra*. The superoxide anion scavenging abilities of extracts were determined as: stems > leaves > rachis > root

In the PMS/NADH-NBT system, the reduction of NBT with NADH mediated through PMS under the aerobic conditions was inhibited upon the addition of 50% ethanol extracts from *D. erythrosora*. The addition of PMS to this system provoked the reduction and the reduction of NBT through O_2_^-^ occurred in the reoxidation of reduced PMS with O_2_. The extracts from *D. erythrosora *would therefore decrease the steady-state concentration of NBTH (*i.e. *NBT+H^+^+O_2_^-^→O_2_+NBTH·) through scavenging O_2_^-^, leading to the decrease in the rate of the production of formazan via the reaction (*i.e*. 2NBTH·→NBT+NBTH_2_). Hence, the decrease of absorbance at 560 nm with extracts from *D. erythrosora *through the decrease of formazan dye which then indicated the consumption of the generated superoxide anion in the reaction mixture. Figure 4 showed the inhibitory effect of *D. erythrosora *extracts on superoxide radical generation. The scavenging activity of *D. erythrosora *extracts was increased with the increasing concentration. All the *D. erythrosora *extracts from different parts showed strong activities. The superoxide anion scavenging abilities of extracts were determined as: stems > leaves > rachis > root. There is no relationship between IC_50_-values and the total flavonoid contents in leaves, stems, rachis and roots of *D. erythrosora*. It illustrated that there are strong superoxide anion scavengers in *D. erythrosora*. It is worth to separate and identify these components in future.
